# Characterization of buccal cell DNA after exposure to azo compounds: a cross-sectional study

**DOI:** 10.12688/f1000research.25798.2

**Published:** 2020-09-18

**Authors:** Juni Handajani, Urfa Tabtila, Nadia Rully Auliawati, Abdul Rohman

**Affiliations:** 1 Department of Oral Biology, Faculty of Dentistry, Universitas Gadjah Mada, Yogyakarta, 55281, Indonesia; 2 Dental Study Program, Faculty of Dentistry, Universitas Gadjah Mada, Yogyakarta, 55281, Indonesia; 3 Department of Pharmaceutical Chemistry, Faculty of Pharmacy, Universitas Gadjah Mada, Yogyakarta, 55281, Indonesia

**Keywords:** DNA, buccal cell, azo-exposed

## Abstract

**Background:** Azo compounds, containing naphthol and diazonium salts, are synthetic dyes widely used in the batik industry. Azo compounds are considered toxic when they are exposed to human tissue. The purpose of this study was to analyze buccal cell DNA exposed to azo compounds in batik workers.

**Methods: **A cross-sectional study involving 20 male subjects divided into two groups (n=10 group), namely azo-exposed and non-exposed (control group). Inclusion criteria were batik workers of the colouring division who have been exposed to azo for at least 5 years. Buccal cells were taken using cytobrush then DNA were isolated from buccal cell. DNA isolation was done by buccal DNA kit, while the purity and concentration of the DNA was determined using spectrophotometer and electrophoresis.

**Results: **The azo-exposed group revealed higher purity DNA than those in the control group. The purity of the DNA in the azo-exposed group and control group was 0.61±0.93 and 0.21±0.09, respectively, while the concentration of DNA was of 59.02 and 19.35 ng/UL, respectively. The ratio at 260/280 nm was 1.84-1.94 (azo-exposed) and 1.85-1.92 (control). Principal component analysis using the first principle component (PC1) and second principle component (PC2) could successfully classify subjects in the control and azo-exposed groups.

**Conclusion: **Characteristics of DNA could be used as an indication of exposure to azo compounds in workers of batik industries.

## Introduction

The oral mucosa is the first defence against particles entering the body. The oral epithelial mucosa functions to protect the body from chemical, microbial, and physical challenges ^1, 2^. The buccal epithelium is the thickest region in the squamous stratification epithelium. Keratinization is influenced by endogenous or exogenous factors. Exogenous factors include the use of drugs, nutritional factors, and irritant factors, such as plaque and calculus, artificial teeth, and smoking or exposure to other substances ^3, 4^.

The use of azo synthetic dyes and their derivatives, especially those with benzene groups, are increasing in the batik industry ^5, 6^. Azo dyes are compounds characterized with one or more azo functional groups (-N=N-), linked to benzene. They are readily reduced to hydrazines and primary amines. The benzene group in azo compounds is difficult to degrade because it takes a long time ^7, 8^. Chemicals in the batik industry are known to cause irritation to the skin and eyes, and cause interference with the respiratory system ^8^. Azo compounds are also known to be carcinogenic and mutagenic if they are in the environment for a long time, and they are suspected to be a source of disease ^9, 10^.

Exposure to synthetic azo dyes, which are continuously inhaled by batik workers, may cause changes in the oral mucosa. Daily exposure to azo dyes needs to be analysed to assess the possibility of the risk of oral cavity abnormalities, although there have been no reports of batik workers that mention oral cavity abnormalities due to azo exposure. Exposure to azo dyes for more than 5 years in batik artisans has been known to significantly increase the frequency of micronuclei, karyolysis, and pyknosis in buccal mucosal epithelial cells ^11– 13^. In addition, exposure to azo dyes significantly increases the expression of cytokeratin 5 and 19 in the buccal mucosa ^14, 15^. Although previous studies stated that exposure to azo dyes significantly increased the expression of cytokeratin 5 and 19, but clinically it has not shown changes in the buccal mucosa. Until now, there is limited study on the effects of azo exposure on the buccal mucosa. The results of these studies have not yet explained the changes in buccal cell DNA exposed to azo compounds; therefore, the objective of this study was to evaluate the profile of buccal cell DNA exposed to synthetic azo dyes to determine the possibility of cellular damage.

## Methods

### Participants

The method was *cross-sectional* to compare subjects exposed and not exposed at the same time. We conducted the study in batik industries (for exposed group) and non- batik industries (for control group) in Yogyakarta-Indonesia from May to August 2019. The procedure of this study was approved by Research Ethics Committee of the Faculty Dentistry, Universitas Gadjah Mada (Ethical Clearance No.00107/KKEP/ FKG-UGM/EC/2019).

Participants of exposed group were from batik industries in Yogyakarta Indonesia whereas participants of the control group were students and staff at the Faculty of Dentistry, Universitas Gadjah Mada, Indonesia. For the exposed group, batik factories were identified from a list online and information about the study was sent to the manager of the factories (letter No. 5189/UN1/FKG/Set.KG1/PT/2019 from Universitas Gadjah Mada to the factories), who allowed the researchers to interview their workers. For the control group, information about this study was sent to students at our university that asked them to participate in the study. All participants agreed to participate by providing written informed consent.

Information collected from the participants were age, past medical and dental history, occupational history, lifestyle (smoking and alcohol consumption) and if they wore a dental apparatus. Oral Hygiene Index-Simplified (OHI-S) were calculated from calculus index (CI(S)) and debris index (DI(S)): OHI-S = CI(S) + DI(S). Interpretation: 0 - 1.2 is good; 1.3 - 3.0 is fair; and 3.0 - 6.0 is poor ^16^.

Inclusion criteria were aged between 18 and 45 years old (age group most likely to be working), male (to provide continuity among participants), OHI-S status of ‘good’, worked in colouring batik for a minimum of 5 years (for exposed group), and did not work in coloring batik (for control group).

Study size was calculated according to Notoatmodjo ^17^

  $$n=\frac{Z1-\alpha /2P(1-P)}{d}$$
n = number of samples
Z1-α/2 = the Z value at 95% degree of significance is 1.96
P = proportion of subject azo-exposed around 50% (0.5)
d = degree of deviation to population, by 5% (0.05)

Based on the formula, n = 9.8 ≈ 10. In this study, the number of subjects for each group is 10 participants.

### Data collection

Participants were asked to rinse out their mouths first to remove debris in the oral cavity. Buccal epithelial cell harvesting was carried out using the smear method using sterile foam Tipped Swab (Product Code: PW1174, Himedia, India). Swab was done by turning in the direction of at least 360° in the buccal mucosa then put in a microtube. Samples were transported in the microtube with 1x PBS to the lab. Sample collection was carried out at the batik factories for the exposed participants and at the university for the non-exposed participants.

### DNA isolation

DNA isolation was done following the protocol from HiPurA ^TM^ Buccal DNA Purification Kit (Product Code: MB531; Himedia, India). Briefly, the buccal swab sample was placed into a 2.0 ml microcentrifuge tube, 400 µl of resuspension solution was added, and the tube was centrifuged at 14,000 rpm for 5 minutes. The pellet was discarded and the supernatant was transferred to a new collection tube. 20 µl of Proteinase K solution (20 mg/ml) was added to the tube containing the supernatant, and this was vortexed for 10–15 seconds. 20 µl of RNase A solution (20 mg/ml) was added, and the tube was again vortexed for 10–15 seconds. The sample tubes were incubated for 2 minutes at room temperature (15–25°C).

The lysis reaction was done by added 400 µl of lysis solution to the tube, which was vortexed thoroughly for a few seconds to obtain a homogenous mixture. Samples were incubated at 55°C for 10 minutes. For the binding step, 400 µl of ethanol (96–100%) was added to the lysate, which was then mixed thoroughly by vortexing for 5–10 seconds. The lysate was added to the HiElute Miniprep Spin Column (Capped) and samples was centrifuged at 6,500 x g (10,000 rpm) for 1 minute. The flow-through liquid was discarded, and the procedure was repeated with any remaining lysate. A prewash was performed by adding 500 µl of diluted prewash solution to the column and centrifuged at 6,500 x g (10,000 rpm) for 1 minute. The flow-through liquid was discarded and the same collection tube was re-used with the column.

Subsequently, samples were washed by adding 500 µl of diluted Wash Solution to the column and centrifuged at 12,000–16,000 x g (13,000–16,000 rpm) for 3 minutes to dry the column then the flow-through was discarded and a new uncapped 2.0 ml collection tube was placed in the column. DNA Elution was done by pipetting 150 µl of the Elution Buffer directly onto the column without spilling on the sides. The samples were incubated for 1 minute at room temperature and centrifuged at >6500 x g (10,000rpm) for 1 minute to elute the DNA. Storage of the eluted purified DNA was done at 2–8°C for short-term (24–48 hours) or -20°C for long-term storage.

### Evaluation of DNA characteristics

Purity and concentration of DNA buccal cells were characterized using electrophoresis and spectrophotometer. Agarose gel was prepared in concentration of 2%. Agarose (Biotechnology Grade, 1 ^st^ Base, Singapore; 1 gr) was added to 50 ml Tris/Borate/EDTA (TBE) buffer, then put in microwave for around 2 minutes until completely dissolved. After agarose solution was cooled to about 50°C (around 5 minutes), this was poured into a gel tray with the well comb in place and then let sit at room temperature for around 20 minutes until agarose gel had completely solidified. Loading dye 1μl was prepared on parafilm then added 5 µl DNA, aspirated in the micropipette, put into the agarose well. Agarose gel was placed into the gel box (electrophoresis unit), then filled gel box with 1x TBE until the gel was covered. Electrophoresis was run at 100 mA and then visualized with Florosafe DNA Stain (Genetika Science, PT. Genetika Science Indonesia) using a UV transilluminator. Concentration of DNA buccal cell were measured using a spectrophotometer. Purity of DNA was analysed using spectrophotometer at 280 and 260/280 nm.

### Data analysis

Data analysis was performed using stat Shapiro-Wilk and Levene’s test, to see if the data were normal and homogenous. Mann-Whitney U test was used to describe the comparison between azo-exposed group and the control group. Statistical measurement was performed using IBM SPSS Statistics v22. The classification of the azo-exposed group and control group was performed using chemometrics of principal component analysis (PCA) using Minitab version 17. P<0.05 was taken as significant.

## Results

Characteristics of study participants are described in Table 1.

**Table 1.** Demographics of azo-exposed batik workers and control group (non-exposed).

Group	Total, n	Male gender, n	Mean age, years	Oral Hygiene Index-Simplified rating of ‘good’, n	Smoking, n	Alcohol consumption, n	Wearing dental apparatus
Azo- exposed	10	10	27.6	10	3	0	0
Control	10	10	18.1	10	2	0	0

**Figure 1.**
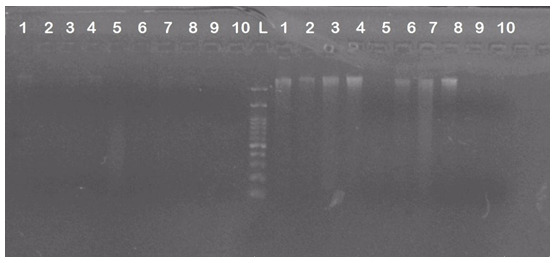
DNA electrophoresis obtained from buccal epithelial cell control (left 1–10) and azo-exposed (right 1–10) from ladder (L).

The presence of high-molecular weight DNA was evaluated by gel electrophoresis and visualized using a UV illuminator. Figure 1 shows that bands for high-molecular weight DNA only appeared for the azo-exposed group, but it did not appear in control group. There was no significant difference between groups for the purity of the DNA at A _280 nm_ (p=0.076), ratio A _260/280_ (p=0.718), or the concentration of the DNA (p=0.076) ( Table 2).

**Table 2.** Mean and standard deviation of the purity and concentration of buccal cell DNA from azo-exposed and control group.

Group	n	Purity of DNA (optical density)		Concentration of DNA (ng/uL)
		A _280_	A _260/280_ (range min-max)	
Azo-exposed	10	0.61 ± 0.93	1.89 ± 0.07 (1.84–2.07)	59.02 ± 87.08
Control	10	0.21 ± 0.09	1.89 ± 0.05 (1.85–1.92)	19.35 ± 4.41

**Figure 2.**
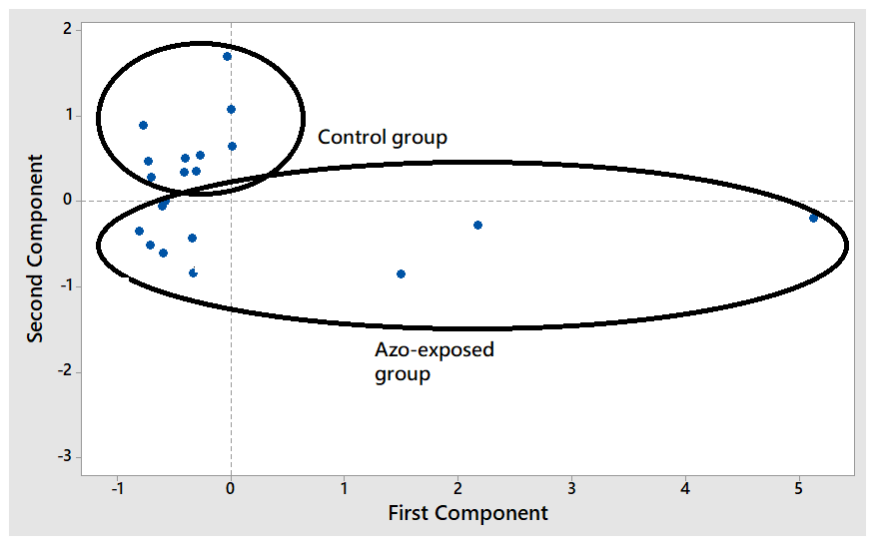
Score plot for the first principal components (PC1) and second principle component (PC2) for participants in the azo-exposed group and control group.

**Figure 3.**
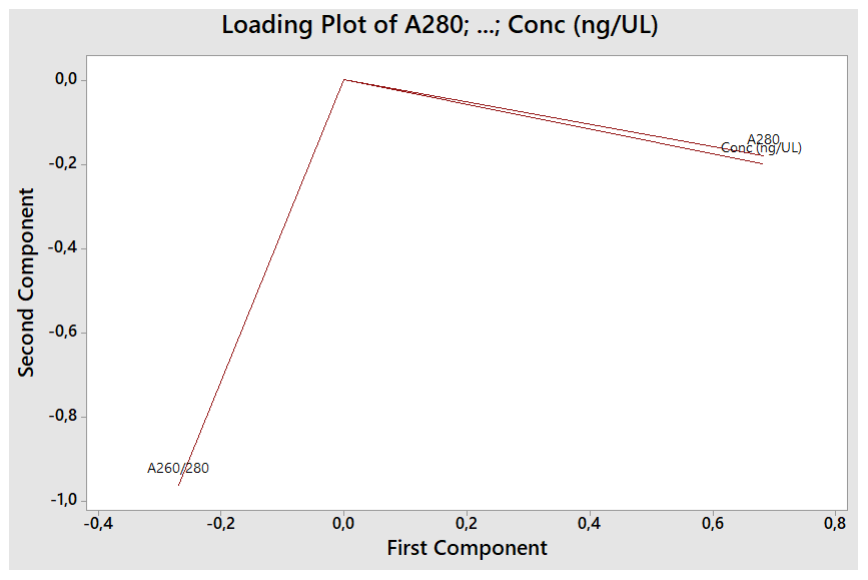
Loading plot of principal component analysis using the purity of DNA at 280 nm, the concentration of DNA, and the ratio of absorbance values at 280 and 260 nm (A _280/260 nm_).

PCA could successfully classify participants in azo-exposed and control groups. The score plot for the ﬁrst principal components (PC1) and second principle component (PC2) is shown in Figure 2. In addition, the loading plot for the evaluation of variables contributing to the separation is shown in Figure 3. The concentration of DNA contributed the most in PCA, as it was the fartherest variable from the initial points (0.0).

## Discussion

The method of this study was done using exfoliative buccal epithelial cells by swab tip or cytobrush then purity and concentration of the DNA was analysed. The exfoliative method was non-invasive. One of the important procedures in the study for DNA extraction was the collection method of the sample. According to Mulot *et al.* ^18^, cytology brushes (cytobrush) are the most appropriate method and provide good quality cell collection compared to mouthwashes, swabs, or collected from saliva.

In the present study, DNA electrophoresis revealed a band for high-molecular weight DNA in azo-exposed group only ( Figure 1). This result indicated that the concentration of the DNA from buccal epithelial cells in azo-exposed group was higher than controls. However, we noticed that not all samples in the azo-exposed group revealed a band. This may be because of the low concentration of DNA in the collected buccal epithelial cells. Another possibility, azo may cause DNA breakage in the human cell. In accordance with previous study, the component azo dye Disperse Orange 1 may cause apoptosis and DNA breakage in HepG2 cell derived from human hepatoma ^19^.

This result was supported by our spectrophotometer measurements ( Table 2), showing that DNA concentration in the control group was lower than in the azo-exposed group. DNA quality may have been affected by collection and isolation methods. This result showed the mean OD 260/280 ratio was 1.89 both in the azo-exposed and control groups, which indicates that the bulk of the proteins were removed successfully. According Desjardins and Conklin that pure nucleic acids typically was in yield a 260/280 ratio of ~1.8 for DNA ^20^.

The standard deviation for the purity of the DNA at A280 nm and the concentration of the DNA ( Table 2) in azo-exposed groups was higher than the mean. This indicates that the purity of the DNA from azo-exposed participants varied, which may be due to exposure of azo that has induced DNA damage. According to Ferraz *et al.* ^19^, the azo dye, Disperse Orange 1, which is used in textiles, induces a frameshift mutation and cytotoxic effect in the human hepatoma cell line HepG2. Mutagenicity was shown by enhanced nitroreductase and o-acetyltransferase, which are important enzymes in mutagenicity. This result was also supported by a previous study that showed that azo dye exposure increases the number of micronuclei, karyolysis, pyknosis, and expression of cytokeratin 5 and 19 in oral epithelial cells ^11– 15^. However, these results have not yet revealed how the mechanism of DNA damage occurs in oral epithelial cells due to azo exposure.

In order to classify participants into azo-exposed and control groups, principal component analysis (PCA) was used. PCA is capable of projecting the initial variable data in reduced dimensions deﬁned by principal components (PCs). The value corresponding to the PC is known as score plot ^21, 22^. PCA was done in this study using three variables, namely the purity of DNA at 280 nm, the concentration of DNA, and the ratio of absorbance values at 280 and 260 nm (A _280/260 nm_). Our results showed that the azo-exposed group could be separated successfully and easily differentiated from control group using PC1 and PC2 score plots ( Figure 2). The loading plot of PCA was performed to evaluate the variables having the most significant contribution to the separation and classification of participants as azo-exposed and controls. The loading plot can explain the projection of variables used during PCA in the same plane as the score plot ^23^. The absolute value of loading in the variables explains the importance of the contribution of each region. Therefore, the further the variables are from the origin of the variable point, the larger the contribution of that variable to the PCA model ^24, 25^. The results of the loading plot indicated that all three variables made a significant contribution to the PCA model.

## Conclusion

Buccal cell DNA of batik workers exposed to azo compounds had higher purity of DNA, concentration of DNA and absorbance ratio at 260/280 than buccal cell DNA of controls (not exposed to azo compounds). Principal component analysis, based on score plot, could successfully classify participants as controls and azo-exposed individuals. The characteristics of DNA could be used as an indication of exposure to azo compounds in workers in batik industries.

## Data availability

### Underlying data

Figshare: Raw data-Dana Masyarakat 2019, https://doi.org/10.6084/m9.figshare.12733055.v5 ^26^.

Data are available under the terms of the Creative Commons Attribution 4.0 International license (CC-BY 4.0).
