# Supplementary material for: Characterization of buccal cell DNA after exposure to azo compounds: a cross-sectional study
Source: F1000Res. 2020 Sep 18;9:1053. Originally published 2020 Aug 27. [Version 2] doi: 10.12688/f1000research.25798.2 (PMC7509593; doi:10.12688/f1000research.25798.2)
Supplement: Peer review history

# Peer review: Reviewer response for version 2

**DOI:** 10.5256/f1000research.29563.r71603

**Published:** 2020-09-22

**Authors:** Boy M. Bachtiar

**Affiliations:** 1 Department of Oral Biology and Oral Science Research Center, Faculty of Dentistry, Universitas Indonesia, Jakarta, Indonesia

In general, the author's response, in my opinion, can be accepted.

 My concern is only regarding the  DNA concentration that had been put into the agarose well. According to the author's response, they put the DNA  in volume of 1uL into the agarose well. However, it is still not clear how much DNA concentration has been in 1uL (volume).

Is the work clearly and accurately presented and does it cite the current literature?

Partly

If applicable, is the statistical analysis and its interpretation appropriate?

I cannot comment. A qualified statistician is required.

Are all the source data underlying the results available to ensure full reproducibility?

Partly

Is the study design appropriate and is the work technically sound?

Partly

Are the conclusions drawn adequately supported by the results?

Partly

Are sufficient details of methods and analysis provided to allow replication by others?

Partly

Reviewer Expertise:

Oral microbiology

I confirm that I have read this submission and believe that I have an appropriate level of expertise to confirm that it is of an acceptable scientific standard.

# Peer review: Reviewer response for version 1

**DOI:** 10.5256/f1000research.28470.r70435

**Published:** 2020-09-04

**Authors:** Boy M. Bachtiar

**Affiliations:** 1 Department of Oral Biology and Oral Science Research Center, Faculty of Dentistry, Universitas Indonesia, Jakarta, Indonesia

Title: I suggest “Risk assessment of DNA damage exposed to azo compound”

**Introduction**

- I suggest, in the introduction section, the author needs to write a strong background in this study. Please add the fundamental reason behind it. For example: whether the author has observed/found that the population (batik worker) were consisting of those with and without a “clinical symptom” of cellular damage.
- Therefore, the objective of this study was to compare the profile of buccal cell DNA exposed to synthetic azo dyes between the batik worker with and without clinical symptoms of cellular damage.  

**Methods**

- I think this study did not need to use control of those who come from not exposed subjects.
- There is no explanation of the concentration of DNA that was put into the agarose well?

 

**Results**

- It would be better if the characteristics of study participants incorporated with pictures showing the buccal mucosa of participants with and without clinical symptoms.

 

**Discussion**

 In general, this section needs to be revised. Please do not repeat the result but discuss it to answer the research question (this needs to clearly explain in the introduction section).

 Specific questions:

 "This result was supported by our spectrophotometer measurements (Table 2), showing that DNA concentration in the control group was lower than in the azo-exposed group. DNA quality may have been affected by collection and isolation methods. This result showed the mean OD 260/280 ratio was 1.89 both in the azo-exposed and control groups, which indicates that the bulk of the proteins were removed successfully."

- In the second paragraph, “DNA electrophoresis revealed a band for high-molecular weight DNA in azo-exposed group”, indicating the DNA concentration was higher in BEC in azo exposed people. What does it mean? I only show the same band derived from either group. How much of the DNA concentration was added into the well of agarose?
- Similarly, “This result was also supported by a previous study that showed that azo dye exposure increases the number of micronuclei…………, …………,…However, the author needs to explain what is the obtained data they have to support this statement.
- Also, ……… ”not all samples in the azo-exposed group revealed a band.  The author argues that the unrevealed band could be due to low concentration of DNA in the collected buccal epithelial cells”. I suggest, that they need to explain this result with the possibility of the relationship between the unrevealed DNA and azo exposure.

**Conclusion**

- Please revise the conclusion. T should be related to this study’s aim. Particularly, what does the author mean with the “characteristics of DNA”?  It could be used as indication (indicator?) of clinical symptom of azo exposure? The meaning is not clear.

 

**References**

- Some references (no 11, 12, and 13) can be searched.  Please use references that have been indexed in database.

Is the work clearly and accurately presented and does it cite the current literature?

Partly

If applicable, is the statistical analysis and its interpretation appropriate?

I cannot comment. A qualified statistician is required.

Are all the source data underlying the results available to ensure full reproducibility?

Partly

Is the study design appropriate and is the work technically sound?

Partly

Are the conclusions drawn adequately supported by the results?

Partly

Are sufficient details of methods and analysis provided to allow replication by others?

Partly

Reviewer Expertise:

Oral microbiology

I confirm that I have read this submission and believe that I have an appropriate level of expertise to confirm that it is of an acceptable scientific standard, however I have significant reservations, as outlined above.

# Response

**Published:** 2020-09-12

**Authors:** Juni Handajani

**Affiliations:** Universitas Gadjah Mada, Indonesia

Title: I suggest “Risk assessment of DNA damage exposed to azo compound”

Answer:

Thank you for your suggestion.

In this study, we did not know yet the occurrence of DNA damage, but it was to examine the change of DNA characteristics. We need further study to identify possible DNA damage.

**Introduction**

I suggest, in the introduction section, the author needs to write a strong background in this study. Please add the fundamental reason behind it. For example: whether the author has observed/found that the population (batik worker) were consisting of those with and without a “clinical symptom” of cellular damage.

Therefore, the objective of this study was to compare the profile of buccal cell DNA exposed to synthetic azo dyes between the batik worker with and without clinical symptoms of cellular damage.

Answer:

We added in introduction:

Although previous studies stated that exposure to azo dyes significantly increased the expression of cytokeratin 5 and 19, but clinically it has not shown changes in the buccal mucosa. Until now, there is limited study on the effects of azo exposure on the buccal mucosa.

**Methods**

I think this study did not need to use control of those who come from not exposed subjects.

Answer:

In this study we need control to compare between azo-exposed group and without exposed-azo.

There is no explanation of the concentration of DNA that was put into the agarose well?

Answer:

We added explanation:

Loading dye 1μl was prepared on parafilm then added 5 µl DNA, aspirated in the micropipette, put into the agarose well

**Results**

It would be better if the characteristics of study participants incorporated with pictures showing the buccal mucosa of participants with and without clinical symptoms

Answer:

We apologize, we did not take pictures of the buccal mucosa each subject. All subject did not show any clinical symptoms.

**Discussion**

In general, this section needs to be revised. Please do not repeat the result but discuss it to answer the research question (this needs to clearly explain in the introduction section).

Specific questions:

"This result was supported by our spectrophotometer measurements (Table 2), showing that DNA concentration in the control group was lower than in the azo-exposed group. DNA quality may have been affected by collection and isolation methods. This result showed the mean OD 260/280 ratio was 1.89 both in the azo-exposed and control groups, which indicates that the bulk of the proteins were removed successfully."

Answer:

We added explanation:

According Desjardins and Conklin that pure nucleic acids typically was in yield a 260/280 ratio of ~1.8 for DNA.

In the second paragraph, “DNA electrophoresis revealed a band for high-molecular weight DNA in azo-exposed group”, indicating the DNA concentration was higher in BEC in azo exposed people. What does it mean? I only show the same band derived from either group. How much of the DNA concentration was added into the well of agarose.

Answer:

The results from DNA electrophoresis showed band in azo-exposed group while band was not seen in control group. When prepared the electrophoresis, we used loading dye to visualize the DNA. We added 5 µl DNA into loading dye. If we compared to DNA ladder, a band indicated that concentration in azo-exposed group is higher than in control group.

Similarly, “This result was also supported by a previous study that showed that azo dye exposure increases the number of micronuclei…………, …………,…However, the author needs to explain what is the obtained data they have to support this statement.

Answer:

The results of this study have not yet revealed how the mechanism of DNA damage occurs in oral epithelial cells due to azo exposure even previous study showed there was some alteration in the buccal cell e.g. increases the number of micronuclei, karyolysis, pyknosis, and expression of cytokeratin 5 and 19 in the epithelial cell may be due to dye exposure.

Also, ……… ”not all samples in the azo-exposed group revealed a band.  The author argues that the unrevealed band could be due to low concentration of DNA in the collected buccal epithelial cells”. I suggest, that they need to explain this result with the possibility of the relationship between the unrevealed DNA and azo exposure.

Answer:

We added explanation:

Another possibility, azo may cause DNA breakage in the human cell. In accordance with previous study, the component azo dye Disperse Orange 1 may cause apoptosis and DNA breakage in HepG2 cell derived from human hepatoma

**Conclusion**

Please revise the conclusion. T should be related to this study’s aim. Particularly, what does the author mean with the “characteristics of DNA”?  It could be used as indication (indicator?) of clinical symptom of azo exposure? The meaning is not clear.

Answer:

The result of this study was a preliminary study, and we have successfully to classify participants as controls and azo-exposed individuals based on score plot. We also characterized DNA of buccal cell according purity and concentration DNA, and absorbance ratio at 260/280.

**References**

Some references (no 11, 12, and 13) can be searched.  Please use references that have been indexed in database.

Answer:

We revised some references by adding link.

# Peer review: Reviewer response for version 1

**DOI:** 10.5256/f1000research.28470.r70437

**Published:** 2020-09-04

**Authors:** Futoshi Nakazawa

**Affiliations:** 1 Department of Oral Microbiology, School of Dentistry, Health Sciences University of Hokkaido, Tobetsu, Japan

In this study, authors analysed buccal cell DNA exposed to azo compound in batik workers. And, they showed that the characteristics of DNA could be used as an indication of exposure to azo compound in workers of batik industries.

 Although the number of subjects, 10 for each group, was small, the authors performed statistical comparisons. And they provided sufficient Tables and Fig.s for the conclusion. Also, descriptions of Introduction, Methods, Results and Discussion were reasonable. Therefore, I think that this article is acceptable as Brief Report.

Is the work clearly and accurately presented and does it cite the current literature?

Yes

If applicable, is the statistical analysis and its interpretation appropriate?

Yes

Are all the source data underlying the results available to ensure full reproducibility?

Yes

Is the study design appropriate and is the work technically sound?

Yes

Are the conclusions drawn adequately supported by the results?

Yes

Are sufficient details of methods and analysis provided to allow replication by others?

Yes

Reviewer Expertise:

Oral Microbiology

I confirm that I have read this submission and believe that I have an appropriate level of expertise to confirm that it is of an acceptable scientific standard.
